# Anthropomorphism and Its Adverse Effects on the Distress and Welfare of Companion Animals

**DOI:** 10.3390/ani11113263

**Published:** 2021-11-15

**Authors:** Daniel Mota-Rojas, Chiara Mariti, Andrea Zdeinert, Giacomo Riggio, Patricia Mora-Medina, Alondra del Mar Reyes, Angelo Gazzano, Adriana Domínguez-Oliva, Karina Lezama-García, Nancy José-Pérez, Ismael Hernández-Ávalos

**Affiliations:** 1 Neurophysiology, Behavior and Animal Welfare Assessment, DPAA, Universidad Autónoma Metropolitana (UAM), Unidad Xochimilco, Mexico City 04960, Mexico; azdeinertcg@gmail.com (A.Z.); alondra.reyes.105@gmail.com (A.d.M.R.); mvz.freena@gmail.com (A.D.-O.); kikislezama@hotmail.com (K.L.-G.); nancy.jopz@gmail.com (N.J.-P.); 2 Department of Veterinary Sciences, University of Pisa, 56124 Pisa, Italy; chiara.mariti@unipi.it (C.M.); giacomo.riggio@phd.unipi.it (G.R.); angelo.gazzano@unipi.it (A.G.); 3 Facultad de Estudios Superiores Cuautitlán, Universidad Nacional Autónoma de México (UNAM), Cuautitlán Izcalli 54714, Mexico; mormed2001@yahoo.com.mx (P.M.-M.); mvziha@hotmail.com (I.H.-Á.)

**Keywords:** attachment, behavior, emotions, human–animal interaction, pet clothes, health, malnutrition, zoonoses

## Abstract

**Simple Summary:**

Anthropomorphism refers to the practices in which humans attribute human emotional and behavioral features to non-human animals and objects. For some people, this represents a means to reinforce the human-animal connection, display empathy towards their companion animals, and show care and interest in their well-being. However, some anthropomorphic behaviors towards pets are often driven by temporary fashions that may have a detrimental effect on animal welfare, both physically (e.g., dermatological, orthopedic, and nutritional diseases) and emotionally (e.g., fear, anxiety, aggressiveness). Not less importantly, in some cases, they may pose a risk for public health (e.g., the transmission of zoonotic diseases). This article focuses on the adverse physiological and behavioral effects that may derive from anthropomorphism in order to understand the magnitude of the repercussions on the welfare of companion animals.

**Abstract:**

Anthropomorphic practices are increasing worldwide. Anthropomorphism is defined as the tendency to attribute human forms, behaviors, and emotions to non-human animals or objects. Anthropomorphism is particularly relevant for companion animals. Some anthropomorphic practices can be beneficial to them, whilst others can be very detrimental. Some anthropomorphic behaviors compromise the welfare and physiology of animals by interfering with thermoregulation, while others can produce dehydration due to the loss of body water, a condition that brings undesirable consequences such as high compensatory blood pressure and heat shock, even death, depending on the intensity and frequency of an animal’s exposure to these stressors. Malnutrition is a factor observed due to consumption of junk food or an imbalance in caloric proportions. This can cause obesity in pets that may have repercussions on their locomotor apparatus. Intense human–animal interaction can also lead to the establishment of attachment that impacts the mental state and behavior of animals, making them prone to develop aggression, fear, or anxiety separation syndrome. Another aspect is applying cosmetics to pets, though scientific studies have not yet determined whether cosmetic products such as coat dyes, nail polish, and lotions are beneficial or harmful for the animals, or to what extent. The cohabitation of animals in people’s homes can also constitute a public health risk due to infectious and zoonotic diseases. In this context, this paper aims to analyze the adverse effects of anthropomorphism on the welfare of companion animals from several angles—physiological, sanitary, and behavioral—based on a discussion of current scientific findings.

## 1. Introduction

The term anthropomorphism arises from the Greek anthropos (human) and morphe (form, appearance). It is defined as the act of attributing human characteristics, intentions, motivations, and emotions to non-human animals or objects [1]. An example of this is the teleological ideas that are often linked to anthropomorphic explanations where people confer human-like traits to entities such as divinities the Christian God in 1753. In fact, it may target inanimate natural and artificial objects, natural phenomena, plants, and most commonly, animals [2]. When humans anthropomorphize animals, they attribute to them their own traits, emotions, or intentions. Charles Darwin described this in detail in 1872 [3], pointing out the natural tendency of some people to describe non-human animals as “humanlike” beings. For instance, people may show a greater interest in domestic animals than in insect’ welfare because the latter do not express behaviors similar to humans [4]. On the other hand, this can also be observed in machines such as humanoid robots that represent a technical aspect of anthropomorphism, created with a human-like appearance that could influence the thinking that robots can feel and think like a person [5]. In animals, the tendency to transfer this bonding and attachment may favor or compromise the latter’s welfare.

In extreme cases, non-human animals may be considered “small” or “modified” humans [2], and human needs may be projected onto them. However, this practice may lead to misinterpretation of the actual intentions, motivations, and emotions behind an animal’s behavior, such as believing that a cat is hungry because it meows in front of the refrigerator, that a dog barks to express its desire to play [6], or that Barbary macaques (*Macaca sylvanus*) who bare their teeth are smiling when, in reality, it is a threat signal. In some cases, misinterpretation of animal behavior may trigger intense human–animal conflicts [2].

Often, anthropomorphic behavior is not supported by scientific knowledge, but rather by the human intrinsic need to relate with someone that is easily understandable and that easily understands us. This may lead to interpretative biases of the animal’s actual state, which are often aimed to satisfy the human need for a certain type of relationship, rather than trying to acknowledge, recognize and appease the animal’s actual emotions, motivations, and intentions [7]. This form of anthropomorphism towards companion animals was accentuated in the 20th century when attributions of this kind emerged naturally and unconsciously as people began to form close bonds with animals that show greater morphological similarity to humans, including companion animals as well as those that have an external physical resemblance to humans, such as apes and monkeys [7].

Urquiza-Haas and Kotrschal [6] attribute anthropomorphizing actions to the biophilic nature of human beings; that is, an implicit connection with animals and, more broadly, with nature in general. They add that animals with phylogenetic, appearance, and behavioral similarities to humans are the ones that tend to be anthropomorphized. This would explain why people anthropomorphize domestic animals, especially the ones with which they maintain close relationships (e.g., pet dogs), that have a childlike appearance, or that present external anatomical structures that facilitate affiliation with humans and produce a desire to protect them. However, this affiliation with domestic animals, dogs above all, also has a biological component that resides in the feelings of fondness that humans often feel towards dogs because of their large round eyes, capacity to gesticulate, and the way they use their limbs to scratch the ground or cover their face [8], all of which generate human empathy. Kaminski et al. [9] support this argument by demonstrating how, through ancestral human–canine interactions, domestic dogs have developed human-like facial expressions. For example, the faculty of retracting the levator anguli oculi medialis (movement AU101) allows dogs to move their eyebrows in a way that simulates the human expression of sadness, so they can adopt an appearance equivalent to that of a child by making their eyes seem larger. This triggers caring behaviors in adults. 

Other reasons why humans perform anthropomorphic behaviors with animals include the fact that the way an animal feels and perceives its environment activate the same brain structures in the limbic system and cortical areas [6,10], making it easy to attribute emotions to animals due to similarities to human facial expressions. However, depending on the emotion and species, different regions of the face may participate, possibly intensifying human responses to animals’ emotions. In this regard, Urquiza-Haas and Kotrschal [6] found that when people observe an animal in a state of distress the same brain areas are activated as when witnessing distress in humans, so observing suffering in humans and dogs activates the medial prefrontal cortex, inferior frontal gyrus (IFG), and anterior insula, though it seems that some humans may have greater emotional responses to animal suffering than those triggered by other people’s pain. This is one foundation of empathy: it is easier for people to attribute human emotions to non-human animals when the latter manifest behaviors or signs similar to those of human expressions. 

The need for affect or acceptance in a social group is a basic condition of all gregarious animals, so it is common to perceive that companion animals “make humans happy” as a result of their ancestral coexistence. Undoubtedly, among all non-human animals, dogs enjoy a special consideration by people, who often regards them as an integral part of human society, or even as family members. This preferential status of dogs within human society has probably been fostered by the increasing knowledge on the positive physiological and emotional effects that they may have on people they interact with [11]. In fact, a whole series of human emotional satisfiers may converge to the figure of the companion animal. Díaz-Videla [12] observed that the tendency to anthropomorphize can be propelled by several factors: a need for control, loneliness, satisfaction of one’s social needs, and emotional attachment to non-human companions.

Today, small species play a particularly important role in the lives of many people by generating a human–animal bond characterized by an acceptance and treatment similar to that of family members [13]. Some dogs and cats are treated like children or surrogate friends, perhaps due to the impossibility to procreate or as a consequence of elective parentage [14]. In a positive light, this can influence the decision of dog or cat owners to invest in expensive treatments to keep their non-human companions alive and healthy. However, the humanization of animals has endorsed a whole range of apparatuses, accessories, toys, entertainments, strollers, and innumerable other products: breath-freshening foods, jewelry, float coats, pet cologne, designer clothes, fashions, prams, diapers, shampoos, nail polish, coat dyes, birthday cakes, and shoes, to name just a few that not always respond to animals biological needs [15].

This suggests that, nowadays, pet-owner relationships are often based on the transfer of anthropomorphic features to dogs or cats [16]. Problems may arise when human behavior becomes incompatible with the animal’s needs and, consequently, jeopardizes its welfare, such as when owners feed their pets with foods that alter their natural diet and their metabolism [17]. However, there is some scientific support for the notion that anthropomorphism encourages animal welfare through an increased interest in animal rights and positive thinking towards adoption and pro-animal movements [18]. Today, a growing number of dog and cat owners commonly perform anthropomorphic behaviors. This paper aims to analyze, in light of the current scientific literature, the physiological, behavioral, and public health effects of anthropomorphism that may negatively or positively affect animal welfare. 

## 2. Clothing and Its Effect on Thermoregulation

The skin performs multiple metabolic functions related to thermoregulation, sensorial perception, and protection. This organ is made up of the epidermis, dermis, and hypodermis. The dermis contains the so-called annexes; that is, hair follicles and sebaceous and sweat glands, while the appendices hold the nails. Dog’s skin pH is the highest of all animal species, ranging from 6.2 to 8.6, with an average of 7.52 [19]. Because of the complexity of the functions that the skin performs, dressing dogs can have adverse effects that compromise their welfare by, for example, forming a barrier that may impede or block adequate thermoregulation and alter the balance between heat gain and loss that regulates body temperature. Textiles also raise moisture levels in the skin and may increase adhesion between the cloth and the animal’s skin, producing discomfort or even cutaneous lesions. In cats, chafing between cloth and skin can be a cause of sensory discomfort [20].

Dressing pets is now an everyday practice, as human families take their companion animals along with them in clothes or costumes that often allude to fictional characters. The risk of compromising their welfare and the health of the pet’s skin is greater when owners feed their dogs prior to, or during a walk, because the metabolic digestion process generates caloric energy that irradiates throughout the body and increases even more due to the exercise performed. Maintaining correct cellular function requires a basal metabolic rate [21], however, dogs are taken for walks even in sunny and hot weather conditions which determine the absorption of a greater caloric load, that ultimately needs to be eliminated. Since dogs lack sweat glands, heat loss mainly occurs through panting, which helps lower body temperature through the evaporation of water from the primary airways. To a lesser extent, heat loss also occurs with the passage of liquids from the rich vascular network of the dermis through the skin [19]. 

When animals wear clothes, heat accumulates because physiological mechanisms such as cutaneous vasodilatation and panting are insufficient to dissipate it and maintain a stable body temperature. If heat builds up quickly it can cause heatstroke, or even death, in less than an hour [22]. This reaction can be exacerbated when factors such as the hot weather, in addition to clothing, increase a dog’s body temperature and activates the autonomous nervous system (ANS) and its thermoregulatory mechanisms to effectuate peripheral vasodilatation for heat dissipation through furless structures such as limbs and paw pads [23,24,25]. When this mechanism acts inefficiently the dog’s body temperature will increase [26].

**Figure 1:**
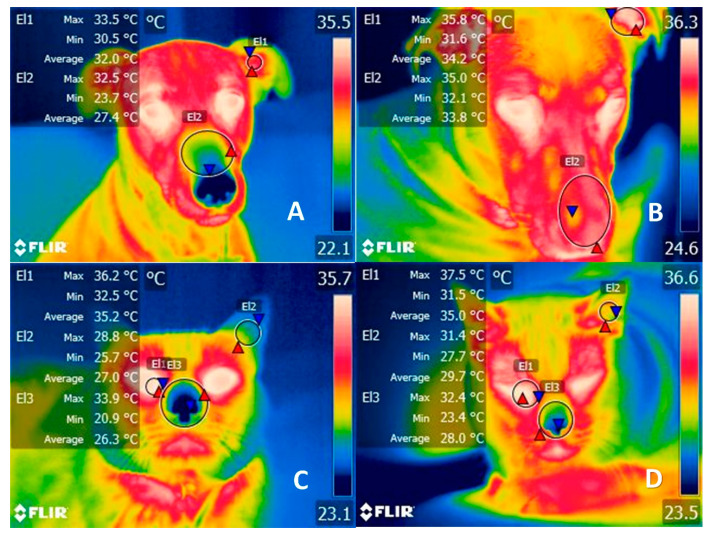
Comparison of the thermal response of clothing in dogs and cats. (**A**) Two year-old female dog without a sweater. The region of the auditory canal (El1) has a temperature range between 33.5 °C (red triangle) and 30.5 °C (blue triangle), while in the nasal region (El2) there is a maximum temperature of 32.5 °C (red triangle) and a minimum of 23.7 °C (blue triangle) before wearing a sweater. (**B**) Thermal pattern of the same canine 8 h after dressing. The increase in maximum temperature by 2.3 °C (red triangle) can be seen in both the auditory canal and the nasal region, although there were no changes in the lacrimal caruncle. (**C**) Cat without a sweater. The facial region of an 8-year-old Mexican domestic breed cat is displayed, where the lacrimal caruncle (El1), ear canal (El2), and nasal region showed maximum temperatures of 36.2 °C (red triangle), 28.8 °C, and 33.9 °C, respectively, before clothing. (**D**) Thermal pattern of the same feline 8 h after dressing. The tear caruncle (El1) showed an increase of 1.3 °C (red triangle), while in the ear canal (El2) and the nasal region the temperature increased by 2.7 °C and 1.7 °C (El3), respectively. Both events represent the effect that the use of sweaters has on the thermoregulation of animals by increasing their body temperature. During hot or controlled climates, such as inside a house, hyperthermia can be exacerbated and cause an increase in core temperature by 2 °C.

In these cases, clothing operates as an additional physical barrier against heat dissipation. When heat does not dissipate, the increase in body temperature accelerates the metabolic rate, which may, in turn, lead to hyperthermia [27]. In Figure 1, thermograms of a dog and cat, with and without clothing, are compared to show the increase in temperature in different thermal regions (ear canal, nose, and lacrimal caruncle) due to the altered thermoregulation. When core temperatures reach 39 °C in dogs and 39.5 °C in cats, thermolysis mechanisms are activated (panting, increased peripheral blood flow) to prevent possible complications such as cerebral edema [26]. Contrary to common belief, the nostrils, rather than the tongue, play a primary role in heat dissipation. This is because the form of the dog’s cornets provides a broad, highly vascularized surface that allows them to lose heat quickly and efficaciously. Canines also have a lateral nasal gland that aids cooling by evaporation, but when used for prolonged periods this mechanism alters the animal’s body water percentage. Therefore, the mechanisms responsible for maintaining thermal neutrality may ultimately affect the animal’s blood pressure, as seen in temperatures above 38 °C, where dogs registered a maximum mean systolic value of 136 mmHg [26].

Hyperthermia due to exercise, high environmental temperatures, or clothing, can cause inflammatory and hemostatic processes (such as coagulation cascade and endotoxemia) that can eventually lead to disseminated intravascular coagulation (DIC) and the systemic inflammatory response syndrome (SIRS). Both usually progress to multiple organ (kidney, brain, skeletal muscle, and liver) dysfunction syndrome (MOD) [22,28], resulting in heatstroke. Brachycephalic breeds such as pugs, boxers, and bulldogs, among others, are especially predisposed to respiratory distress derived from heatstroke because they have stenotic nares, elongated soft palates, and hypoplastic tracheas that perform evaporation inefficiently [22]. It is, then, of primary importance to consider that clothes in dogs can interfere with their thermoregulatory system [23].

**Figure 2:**
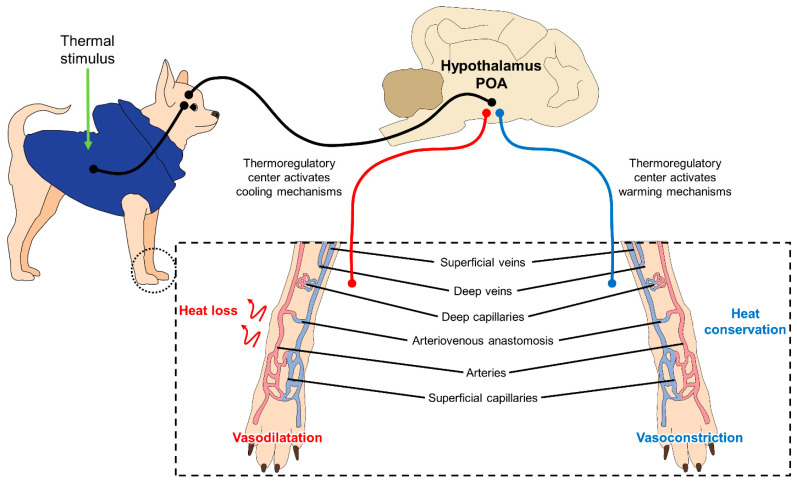
Mechanism of peripheral thermoregulation. The POA responds to a thermal stimulus (cold and hot) activating the thermoregulatory centers located in the hypothalamus. This center promotes mechanisms to dissipate heat or maintain heat through changes in the microcirculation of the skin. When the POA activates cooling mechanisms due to a hot environment, vasodilatation of the superficial capillaries and blood vessels on the limbs produces a heat loss and a decline of the body core temperature. Contrary, when POA activates warming mechanisms in cold settings, the heat conservation, to increase central temperature, is performed by vasoconstriction of these superficial capillaries to shift blood flow to critical organs. POA: preoptic area.

This process of thermoregulation is illustrated in Figure 2, where the superficial blood vessels (capillaries) contribute to heat loss and heat retention through vasodilatation and vasoconstriction, respectively [29]. 

**Figure 3:**
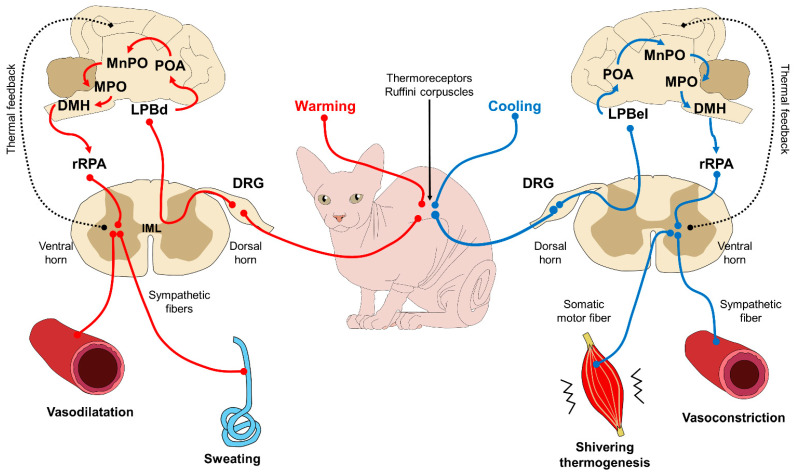
Control mechanisms to regulate body temperature via feedback. Thermoreceptors (Ruffino corpuscles) located in the periphery of the skin sense thermal inputs that are processed by different pathways, depending on the sensory information. The path from the left represents a situation where thermoreceptors read warm or hot stimuli. This input is conducted to the DRG of the spinal cord and projected to superior brain centers. The neuronal axons located in the LPBd are activated by warming stimulus, and project to the POA and other structures within the POA, such as MnPO and MPO. Before returning to the spinal cord, the input goes through the DMH, the rRPA, and reaches the ventral horn, where sympathetic fibers act on blood vessels and glands to dissipate heat by vasodilatation and sweating. The cooling pathway from the right follows almost the exact pattern, except that when a cooling signal is perceived from the thermoreceptors, the information reaches the POA through LPBel (instead of the LPBd). Additionally, the efferent responses employ sympathetic fibers for the vasoconstriction of the skin vessels to conserve heat, and somatic motor fibers start the thermogenesis in the muscles by shivering. When the core temperature returns to thermal neutrality, these mechanisms are regulated with feedback to hinder their influence on the vasculature and other structures. DMH: dorsomedial hypothalamus; DRG: dorsal root ganglion; IML: intermediolateral cell column; LPBd: lateral parabrachial nucleus (dorsal subregion); LPBel: lateral parabrachial nucleus (external lateral region); MPO: medial preoptic area: MnPO: median preoptic nucleus; POA; preoptic area; rRPA: rostral raphe pallidus nucleus.

This caloric exchange is triggered by the central nervous system (CNS) and, more specifically, the hypothalamus, in response to the thermal signaling of peripheral receptors (called Ruffini corpuscles) that perceive hot or cold stimulus (see Figure 3). Under certain conditions, and only upon medical recommendation, clothes may protect against hypothermia in cold or freezing environments when shivering thermogenesis is not enough to maintain homeostasis. In all other circumstances, the practice of dressing these animals is counter-indicated.

## 3. Restricted Mobility and Consequences for the Locomotor Apparatus

**Figure 4:**
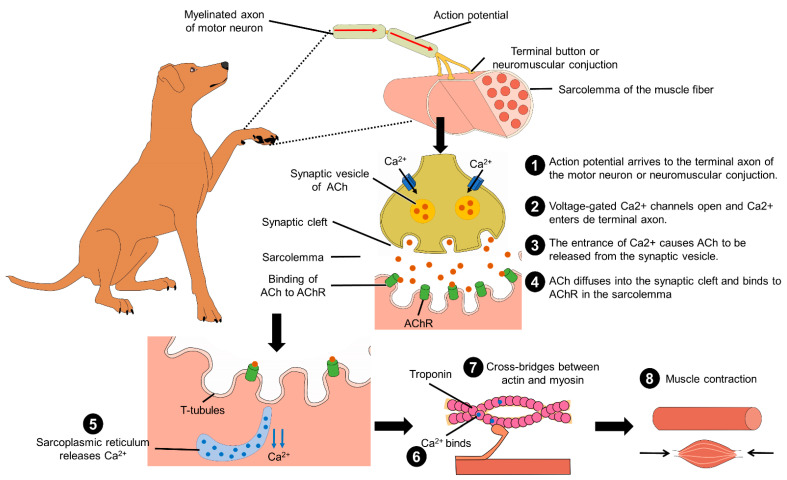
Biochemical process for muscle contraction and movement. In the myelinated axon of motor neurons, the action potential arrives at the neuromuscular conjunction of the sarcolemma. This produces the entrance of Ca^2+^ by opening the voltage-gated Ca^2+^ channels, and the increment of intracellular Ca^2+^ causes ACh to be released from the synaptic vesicle to the synaptic cleft. In the sarcolemma of the muscle, ACh binds to AChR, the action potential travels along the T-tubules. Later, the sarcoplasmic reticulum releases Ca^2+^ that will bind to troponin and produce the cross-bridge between actin and myosin. This interaction leads to muscle shortening and contraction. ACh: acetylcholine; Ca^2+^: calcium.

Another anthropomorphic practice when treating a dog like a child consists of impeding their physical activity and movement; for example, holding pets on one’s lap, carrying them in one’s arms or school bags, or transporting them in strollers designed for babies, for long periods. These practices can affect the behavior and welfare of companion dogs, by reducing their freedom of movement and consequently their ability to control environmental stimuli. This may lead to the development of emotional disorders, such as phobias and anxiety. Furthermore, unnatural postures may also have negative physical consequences. When their limbs are flexed dogs may feel a momentary discomfort and over time, they may develop a condition called biomechanical and metabolic syndrome [30]. When animals move naturally, they do so at different speeds and with distinct forms of locomotion—walking, trotting, running—to exercise their musculoskeletal system, which has three main functions: (1) to generate movement and maintain a correct posture through repeated contractions; (2) to store amino acids so that they are available for general metabolism; and (3) to provide carbon to the liver for gluconeogenesis and the production of glucose that is required for its energy needs. In addition, movement requires certain biochemical processes at the muscular level (Figure 4). These processes are affected when anomalies in muscular structure or function exist [31]. 

A dog’s locomotion consists of a cycle, in which each of its four limbs makes a step, and two phases, called swing and stance. Locomotion has been studied by various techniques, including kinematic analyses [32]. Musch et al. [33], for example, evaluated blood flow in the hind limbs during progressive exercise in 25 untrained, mestizo dogs using radioactive microspheres. They found that intensifying exercise increased blood flow in muscles of the limbs (gracilis, gastrocnemius, semimembranosus, and semitendinosus), as well as in the myocardium. Under conditions of maximum activity, blood circulation was greater in the semimembranosus muscle and lower in the semitendinosus muscle (342 and 134 mL^−1^ 100 g of tissue^−1^ min^−1^, respectively). In a related study, Tøndevold and Bülow [34] analyzed blood irrigation to the bone during exercise, observing that perfusion rates increased by over 50% (from 1.6 to 2.5 mL 100 g of tissue^−1^ min^−1^) in the cortical, femoral, and tibial bones of the dogs during exercise. Additionally, blood flow in the trabecular bone of the femoral head increased from 12.6 to 20.6 mL 100 g tissue^−1^ min^−1^. These results suggest that dogs must perform physical activity to increase osseous density and develop muscle tone. 

Sabanci and Ocal [35] mention that lack of movement can cause muscular atrophy (i.e., reduced muscle mass) that may begin within the first 72 h of inactivity. Atrophy affects the muscles that cross only one articulation more than those that cross two or more joints. Similar to these authors, Thomas [36] stated that muscular atrophy is common in dogs and that these animals often try to compensate for the loss of muscle tone by transferring weight to the other limbs to relieve the pain characteristic of this condition. This affliction is observed clinically as generalized weakness, thinning of the affected zone, lethargy, or movement-related apathy due to pain and muscular flaccidity. 

When mobilization of a dog’s limb is impeded, atrophy and muscle damage can be caused by disuse [37], and also orthopedic disease and osteoporotic fractures can occur due to an increase in bone loss and decreased physical activity [38,39].

But limiting an animal’s movement affects not only its musculature, but also degrades cartilage and reduces the matrix composed of chondrocytes, water, collagen, and proteoglycans. The latter provides a smooth surface and distribute the force over the bones, allowing joints to move freely. Collagen functions to maintain the form of the cartilage by impeding deformation [40], but prolonged immobilization can damage cartilage. Studies have demonstrated that restricting movement in young dogs for just 3–11 weeks reduces the amounts of proteoglycans by 13–60%, and their synthesis by 40–60% [40]. For all these reasons, organisms must maintain constant movement to stimulate the chondrocytes through non-abrupt motions, because cartilaginous zones are avascular and are not innervated, so they cannot regenerate. The ideal way to keep cartilaginous structures healthy and integral is via motor activity [41]. For a return to activity after a prolonged period of inactivity, veterinarians recommend a smooth, gradual reactivation over time to avoid repeated overload that could damage the softened cartilage [40,41].

## 4. Exercise in Inadequate Places and Injuries

Another widely diffused practice is making dogs wear shoes made from various materials to protect their paw pads and prevent erosion of the soles of their feet. However, it is important to understand that dogs’ paw pads are histologically predisposed to endure contact with abrasive surfaces, thanks to their thick corneous stratum. In addition, the superficial fascia of the paw pads contains numerous adipocytes, while the granular layer of the epidermis of the paw pads has a thickness of eight cells, compared to just two in skin covered with fur. In some cases, the stratum lucidus is present in this zone, as it is in the nose. This structure is made up of several layers of non-nucleated keratinized cells and cytoplasmatic organelles. The cytoplasm contains keratin, phospholipids, and eleidin (a protein similar to keratin) but lacks hair follicles and sebaceous glands. There are numerous atrichial glands in the inferior dermis and subcutaneous tissue [19]. These structures buffer wear and tear and prevent injuries to the paw pads. The epidermal surface of the paw pads differs among companion animals, as it is smooth in cats, but papillary and irregular in dogs [42]. Because these are exposed parts of the animal’s body, an external protector such as shoes can cause tissular injuries due to continuous chafing. Hence, making dogs wear shoes should be considered not only unnecessary but also potentially damaging. Indeed, it is important, especially with dogs, to avoid exercise on highly abrasive ground and at high temperatures, such as long walks on the hot concrete or asphalt of city streets, as erosion, heat, or trauma can injure their paw pads, not to mention the risk of heatstroke [43].

In light of this, the suggestion is that owners take their dogs for walks in areas that are less hostile after evaluating the ambient temperature and type of surface. If this is difficult due to the owner’s working hours, then the so-called “5-s test” should be used. This consists in placing the back of one’s hand on the concrete or asphalt for five seconds. If the person cannot withstand the temperature for those 5 s, then her/his dog will not be able to either. Under these conditions, it is better to abstain from walking. If in these conditions the owner decides to put shoes on the dog’s paws, the heat from the pavement will surely pass through and affect the pet’s paw pads [43].

An associated anthropomorphic practice is subjecting dogs to long baths in tubs, but this softens and thins the skin of the paw pads, increasing sensitivity to all types of injury [43]. Instead of putting shoes on their dogs, owners should simply make sure that all activities are performed in accordance with the biology of this species and monitor environmental conditions to avoid harming their pet’s health and physical integrity.

## 5. Alimentary Modifications and Effects on the Organism

Dogs and cats are carnivores by nature. This means they have a predilection for products of animal origin over vegetable foods [44]. A dog’s taste for certain foods develops between 4–7 weeks of life when its mother teaches initial alimentary habits. Dogs prefer animal proteins, so the viscera, liver, and raw intestines are much more attractive to them than cooked meat. They also prefer fats of animal origin over vegetable sources. Their favorite meats seem to be, in descending order, beef, lamb, horse, and poultry. They also have a taste for moist foods (40–60% of HR) over dry foods [44]. In their natural state canines do not ingest sugar, so they can only acquire a taste for sweet flavors during lactation. Despite these traits, in recent years many owners of domestic animals have chosen to change their pets’ diet to one similar to human alimentation by projecting their philosophical ideas or preferences onto their dogs and cats. Observations show that they feed dogs junk food, candies, chocolate, ice cream, cake, and sodas, among other items, none of which are healthy for humans, much less for companion animals. Dietary modifications can have severe consequences for animal health if they include foods that fail to satisfy the pet’s basics nutritional needs; for example, by eliminating amino acids or essential fatty acids and/or providing inadequate ingestion of total calories. 

Dietary variations are nothing new. It is well known that since the domestication of companion animals biochemical and functional changes in the digestive apparatus of the evolutionary ancestors of dogs-wolves have modified their carnivorous stomach and increased their capacity to digest starch. In fact, consumption of kibble shows that dogs have digestibilities greater than 98% for simple carbohydrates such as monosaccharides (e.g., glucose, fructose), disaccharides (e.g., maltose), sucrose, and lactose, among others [45]. With respect to grains such as rice and corn, figures indicate digestibility levels close to 90% [46]. Studies of dogs have demonstrated that the presence of genes involved in synthetizing digestive enzymes and translation factors of several proteins associated with the digestion of starch differs from that of wild wolves [47]. These findings reveal an evolutionary adaptation in human–animal interaction [48]. Despite this capacity to digest carbohydrates, feeding dogs inappropriate diets with high starch content will considerably worsen health problems. Another factor to consider is that dogs are gluttonous; that is, an innate desire that can lead them to eat several times a day [44]. This can result in problems of obesity due to a high consumption of carbohydrates, especially if coupled with sedentism. This health problem is often diagnosed in clinical veterinary medicine today.

Writing on this form of malnutrition, Van Herwijnen et al. [49] stated that problems such as obesity and malnutrition which reduce the welfare of domestic animals are a consequence of incorrect alimentation. This can range from diets based on bones and raw foods (BARF) that, in the absence of appropriate sanitary handling, can introduce pathogens that affect animal health, to the extreme of giving vegan diets that produce a nutritional deficit in animals, especially dogs and cats that belong to the order carnivores. Malnutrition can also cause metabolic problems. We know, for instance, that it is essential to supplement a cat’s vegan diet with taurine because this important amino acid exists only in meat. This form of supplementation may also be advisable for dogs since studies have determined that deficiencies of nutrients such as taurine and carnitine can cause dilated cardiomyopathy. Research on breeds such as the cocker spaniel, beagle, and golden retriever have shown cardiovascular benefits when animals receive these supplements even when a deficiency of both chemicals remains to be clinically documented [50]. For these reasons, dogs and cats that are fed vegan diets require supplements to remedy these deficiencies [16,51,52,53]. Unfortunately, pet owners are often unaware of these data and unintentionally jeopardize the appropriate functioning of their pet’s organism. 

### 5.1. Malnutrition: Effects on Skin and Fur

Nutritional deficiencies may also be reflected in the skin and fur of pets because these structures have non-specific defense mechanisms and barriers that are frequently affected by malnourishment or the absence of certain nutrients. These factors increase the vulnerability of the skin and fur to contracting diseases. When these natural barriers are crossed, various dermatopathies can develop with specific symptoms, such as reddening, psoriasis, scaling, and alopecia [54]. Sadly, the first signs of skin-related problems usually appear only 2–3 months after implementation of an inadequate diet or one lacking in such nutrients as fatty acids, including linoleic and linolenic acids. Once administered to the organism, these acids can be transformed into arachidonic, eicosapentanoic, and docosahexaenoic acids, whose role is to maintain the structure and function of the skin by helping conserve the architecture of the cell membrane and conferring impermeability to water. But these fatty acids also act as precursors of eicosanoids (such as prostaglandins) that are required to regulate inflammatory processes and blood coagulation [54,55].

Linoleic, linolenic, and arachidonic acids are deemed essential for complete nutrition in various mammal species. However, like other animals, cats and dogs lack the capacity to synthetize linoleic acid, so it must be ingested into the diet. In cats, the low activity of the delta-6 desaturase enzyme in cats means they cannot satisfy their physiological requirements for arachidonic acid by converting linoleic acid. Thus, arachidonic and linolenic acid—two essential nutrients for felines—must be given in food [54]. A prolonged deficit of these essential fatty acids results in alopecia, an added consequence of the lack of other nutrients, such as zinc and vitamins A and B (primarily biotin). Because many pet owners consider raw eggs a food of high biological value for humans, they feed them to their companion animals, but this generates an imbalance in necessary nutrients that can produce alopecia around the eyes and face because eggs contain a protein called avidin that interferes with gastrointestinal absorption of biotin [55]. Unfortunately, clinical signs of this condition may not become evident for a long period (months). They generally begin with slight scaling and loss of brilliance in the fur. The incidence of this deficiency has decreased thanks to the supplementation of fatty acids in commercial diets [54], but this indicates the importance of understanding that human diets are not appropriate for dogs and cats and can cause them dermatological and organic dysfunctions.

The utilization of foods destined for human diets may have other negative consequences. For example, vegan diets have a significant lack of arachidonic acid and taurine. The daily requirement of taurine for cats is approximately 500 mg but can only be obtained from fresh foods of animal origin (meat, liver, heart, brain) [52,53]. Diets based solely on foods of vegetable origin cannot provide this nutrient. Additional research is required to determine the ideal diets for dogs and cats. Marsh et al. [56] analyzed the dermal effect of zinc (23.9 mg MJ^−1^, and/or linoleic acid at a concentration of 3.6 g MJ^−1^), in a completely balanced diet of adult dogs. After nine weeks with a standard diet and the same period of time with the enriched diet, dogs consuming the mineral and the fatty acid improved the appearance of the fur (brilliance (*p* = 0.05), scaling of their fur (*p* = 0.007)). According to these results, a supply of zinc and linoleic acid with commercial diets improves the health of the animals’ skin and fur.

### 5.2. Obesity and Osteoarthritis

Overfeeding alongside a lack of physical activity due to close human–animal relationships can cause obesity, a health problem that is increasing in both frequency and severity. Obesity has serious consequences for animal health, including diabetes, lameness, hypothyroidism, and cardiovascular disease [57]. Genetics or level of activity are elements that can contribute to obesity, but an imbalance in food ingestion when owners provide foods that contain excess energy in proportion to the energy expended is one of the principal reasons. This fosters an accumulation of caloric reserves and adipose tissue that not only increases an animal’s weight but affects its overall physical welfare. Obesity can also be a precursor of the development, or complication, of orthopedic illnesses, hyperlipidemia, cardiovascular disease, urinary and reproductive complications, neoplasias, skin disorders, and difficulties with anesthesia [58]. 

Based on reports from veterinary clinics we know that anywhere from 20–50% of dogs present obesity, due to an excessive number of meals, snacks, and/or rewards given daily. The quality of commercial food (e.g., cheaper brands) offered can also predispose dogs to obesity [58,59,60]. In a study by Bland et al. [61], 153 veterinarians in Australia determined that 97% of the problems of obesity were attributable to nutritional influences (e.g., overfeeding) and owners’ lifestyles (e.g., little physical activity, human obesity). In addition, owners tended to overestimate the ideal weight of their pets and had incorrect perceptions of body condition scores. As a result, they continued to provide diets that were inadequate for the species of dog they owned. 

In conditions of obesity, adipose tissue participates in the secretion of leptin by adipocytes. In normal conditions—without obesity—this hormone, aided by thermogenesis, helps regulate excess calories and allows activation of the hypothalamus to diminish appetite and food ingestion. Plasma levels of leptin in obese dogs are high because they have more adipose tissue and, hence, greater secretion of this hormone. However, this induces resistance to leptin and inhibits activation of the hypothalamus [60]. Other studies have shown that the secretion of leptin affects the chondrocytes and favors the development of articular diseases such as osteoarthritis [62]. Obesity is also characterized by the progressive degeneration of articular cartilage that, in normal conditions, separates two bony surfaces, permitting sliding movements while cushioning mechanical load [63,64,65].

**Figure 5:**
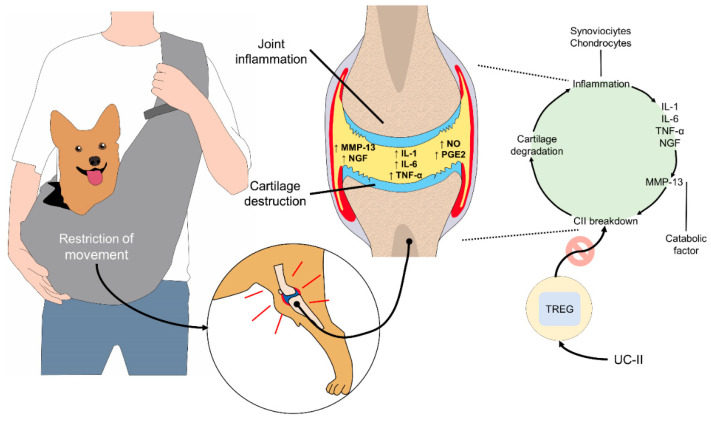
Osteoarthritis mechanism due to restricted movement in companion animals. When a dog or cat is unable to move voluntarily for a long period, cartilage degradation takes place as a result of an inflammatory process in the joints. Synoviocytes and chondrocytes secrete pro-inflammatory substances (IL-1, IL-6, TNF-α), neuropeptides (NGF), mediators (NO and PGE2), and MMPs. Individually, MMP-13 is a catabolic factor that breaks down the CII with the consequent cartilage degradation and loss. This pathophysiology of cartilage damage induces a degenerative joint disease cycle with persistent inflammation in animals. UC-II is a novel pharmacologic alternative that uses immune cells, (TREGs) to block the pro-inflammatory effects. After TREGs recognize the presence of UC-II, these cells secrete anti-inflammatory mediators and promote cartilage repair. CII: collagen type II; IL: interleukin; MMP-13: metalloproteinase 13; NGR: nerve growth factor; NO: nitric oxide; PGE2: prostaglandin E2; TNF-α: tumor necrosis factor alpha; TREG: regulatory T cells; UC-II: undenatured type II collagen.

Cartilage degraded by osteoarthritis is released into the synovial fluid, where phagocytosis occurs, generating inflammation. In consequence, subchondral bone, which is responsible for distributing the mechanical load and nourishing the bone in different stages of growth, may absorb continuous impacts. This can produce pain, limping, and rigidity, conditions worsened by the secretion of leptin, which acts as a growth inhibitor by producing interleukin-1 (IL-1) that, together with the action of the metalloproteinase-9 and -13 matrixes (MMP-9 and MMP-13, degrades cartilage (Figure 5) [60,66,67]. Obese dogs have joints with high levels of mechanical stress due to the load of extra body weight. This predisposes them to develop osteoarthritis. Reports suggest that 15–20% of obese dogs have this condition that most commonly affects the knees and hips [63,64,65].

### 5.3. Malnutrition and Its Effect on Immunity

Pet foods are scientifically formulated to provide the appropriate proportions of nutrients. Currently, prebiotics and probiotics are also incorporated to benefit the microbiome and its role in immune function. The gastrointestinal microbiome is composed of billions of cells that naturally inhabit the digestive tract, from the oral cavity to the rectum. It has diverse benefits for animals, such as facilitating food breakdown and producing metabolites, short-chain fatty acids, secondary bile acids, vitamins, nutrients, and other compounds originating from bacteria [68].

In recent years, diets prepared at home by pet owners have become popular and promote the human-animal relationship. Under rigorous medical guidance and with a detailed understanding of the pros and cons of the foods included, diets of this kind can be considered an alimentary option in specific cases where supplementation is required. In reality, however, many such diets are based on what owners assume is appropriate for the pet or reflect only the alimentary preferences it manifests. Reports indicate that fewer than half of these diets include sufficient amounts of nutrients. The main deficiencies are certain vitamins (E, D, B12), essential fatty acids, and macronutrients (calcium, iodine, selenium, copper, zinc) [69]. An imbalance of macronutrients in poorly formulated diets—as when humans share food with pets, or relative proportions vary—can affect their digestive function. Inadequate diets can, therefore, have significant negative effects on animal welfare, especially certain aspects of the health of dogs and cats, due not only to the incidence of gastrointestinal illnesses, but also allergies, altered buccal health, increased weight, and a predisposition to diabetes and renal diseases, all caused by changes in the gastrointestinal microbiome [68].

## 6. Application of Cosmetics and Their Effects

In many countries, pets have become surrogates for maternity and childcare, but in some cases, this leads to excessive care of companion animals that includes projecting standards of human beauty onto them [70,71]. This tendency has sparked a confrontation between two sectors of the human population: one that supports anthropomorphic practices that promote the use of cosmetic products on pets, the other represented by national and international organizations dedicated to protecting animals and preventing animal cruelty, that seek to prohibit or regulate the use of animals for testing cosmetic products [72].

In this context, practices related to animal hygiene and care have changed from a focus on maintaining animal health to one concerned with developing aesthetic tendencies associated with human concepts of fashion [70]. One practice whose popularity is growing rapidly in developed countries in Asia, such as Japan, Korea, and China, consists of dyeing the fur of pets [73]. Tendencies of this kind are widely disseminated, but many pet owners may be unaware of the harmful effects that the active ingredients in dyes can have on their pets and on human health. Some compounds can damage the skin and fur of dogs and cats and cause lesions to histological structures. Moreover, they present a risk of systemic intoxication if the animals ingest them while performing natural grooming behaviors. One report concerned a dog that accidentally ingested a high concentration of *Lawsonia inermis*—henna—a shrub used worldwide as a cosmetic colorant for dyeing horsehair and human skin, among other items. The use of this chemical with an 8-year-old sterilized female Border Collie that weighed 20 kg caused hemolytic anemia. Likewise, laboratory assays have determined that small amounts of the active ingredient lawsone (2-hydroxy-1,4 naphthoquinone) ingested or applied topically to rats or humans produces toxicity [74]. In this regard, recent studies designed to optimize vegetable dyes for pets specify three factors that affect the product: pH, concentration, and dyeing time [75].

Another harmful aspect of anthropomorphic practices that apply cosmetics to pets is that they can interfere with the animals’ sense of smell. The olfactory capacity of dogs is undeniable and sometimes grossly underestimated because as humans we focus more attention on our visual system. For dogs, however, odors in the environment constitute an extremely effective mechanism of communication. For example, animals exposed to the smell of sweat showed a greater stress response than those that perceive positive or neutral odors. The former also present higher heart rates, seek shelter with their owners, and shy from social contact with strangers [76]. Berns et al. [77] carried out a cohort study of dogs (n=12) using functional magnetic resonance imaging with awake animals, exposing them to five aromas: known human, unknown human, familiar dog, unknown dog, and the dog’s own odor. They were able to determine that activation of the caudate nucleus (associated with positive stimulus) suggested that the dogs positively discriminated the odor of a known and unknown human. This demonstrates the sensibility and importance of the olfactory system in animals. 

In addition, it is important to note that dogs have characteristic odors due to the secretion of chemical substances by glands in their paw pads, ears, and anus. These odors serve as a means of communication with other dogs. Today, pet owners often send their dogs to beauty salons where they are bathed, and colognes, lotions, or perfumes may be applied to mimic the typical odors of pets. Those odors, however, may result in the dogs being considered foreign or strange when they return to contact with conspecifics. Similarly, applying nail polish to dogs is a frequent practice among owners, despite reports indicating that these products may contain harmful substances for animals—and humans—and can produce allergies [78]. Research conducted in the US in the early 2000s found that several nail polish products contained dibutyl phthalate (DnBP) to improve the flexibility of the film, prevent chipping, and conserve color, but animal studies found that DnBP can have toxic effects on reproduction [79].

Gaps in the scientific information available on the effects of applying cosmetics to pets show the need for additional studies in this field because practices of this kind continue to spread in the absence of solid evidence that would allow us to verify if they are harmful to the welfare of the pets involved.

## 7. Effects of Anthropomorphism on Dog Emotions and Behavior

To date, there is no scientific consensus about the effects of anthropomorphism on companion dogs’ psychological well-being. Previous studies suggest that anthropomorphic thinking may result in a more positive attitude towards general animal welfare issues [18]. For example, anthropomorphizing dogs and the empathy that can surge from it, influences the perception of pain and animal suffering, and owners tend to recognize this adverse effect and acknowledge the importance of its mitigation to preserve their welfare [80]. Moreover, empathy and viewing them as species that can develop human-like feelings determine how people treat and care for them [81]. Nonetheless, its practical application in everyday human–animal interactions is often regarded as a possible threat to the well-being of the animals involved.

Surprisingly, the literature on the effects of anthropomorphic practices on the emotional wellbeing of pets is still scarce and the findings are conflicting. A dated study by Voith et al. [82] failed to demonstrate that anthropomorphized pet dogs displayed more problematic behaviors than dogs that were not usually treated like a person. On the contrary, they found that dogs that experienced some anthropomorphic behaviors had fewer behavior problems. However, the majority of the anthropomorphic attitudes this study investigated referred to owners’ spoiling tendencies that do not necessarily entail an actual anthropomorphic thinking, such as sharing the bed or furniture with companion animals, celebrating the dog’s birthdays, sharing food from the table, etc. [82]. For instance, allowing the dog on the bed may not be driven by an anthropomorphic view of the animal, but rather by the owner’s own pleasure or by the assumption that the dog will be more comfortable on the bed than on the floor, which is hard to deny. 

Nevertheless, it is equally undeniable that attributing human mental and emotional states to a dog may lead to anthropocentric misinterpretations of its behavior, which may result in turn to interspecific interactions that may negatively affect the animal’s welfare [83]. A practical example of the risks of dog owners’ anthropocentric attitude is provided by the common belief that dogs are capable of complex feelings, such as guilt [84]. According to Hecht et al. [85], guilt is a self-conscious, evaluative emotion that arises from one’s own perception of having violated an established rule. The most common scenario in which owners attribute such emotion to their pets is that of a dog who, after having been left alone at home and having performed destructive behavior because of anxiety, fear, or boredom, displays submissive and fearful postures when the owner returns. For many owners, these postures are an indication that the dog is aware of having misbehaved during their absence. However, previous studies demonstrated that dogs display “guilty” behavior even when they are not responsible for the undesirable event [86]. Furthermore, findings by Horowitz et al. [87] suggest that what is commonly considered as “guilty behavior” is instead the dog’s response to their owners’ behavior at reunion (i.e., scolding). Furthermore, Hecht et al.’s [85] experiment revealed that the greeting behavior of dogs that transgressed in the owner’s absence does not differ from that of dogs that did not. These results suggest that domestic canine’s “guilty” behavior in the context described does not associate with their awareness of the transgression, but rather with an attempt to appease the owner’s aggressive demeanor during reunion [87]. When interactions of this kind occur repeatedly, the dog may develop anxiety in anticipation of the owner’s return and display appeasement behaviors even in the absence of the owner’s aggressive cues. 

In this context, it is clear how anthropomorphism may have a negative impact on companion dogs’ emotional well-being. This is especially true for those subjects that suffer from separation anxiety disorders, for which the owner’s return should represent the solution to their emotional distress. Unfortunately, many owners do not limit themselves to misinterpreting the dog’s appeasement behavior as an expression of guilt, but they also anthropocentrically assume that the destructive behavior carried out by the dog during their absence is motivated by spite rather than panic [86]. Obviously, these owners are more prone to punish their dogs for their destructive behaviors [88], transforming themselves from a potential source of safety and reassurance to an additional source of distress.

Indeed, this vicious circle may have detrimental effects on the dog–owner relationship and on the quality of the dog attachment bond to the owner, which is mainly determined by the ability of the latter to provide safety in conditions of emotional distress [89,90,91]. The owner’s failure to be a source of safety to their dog may result in the development of an insecure attachment style [90,91,92], which, in the human psychiatric literature, has been linked to a variety of psychopathological disorders, such as anxiety [93,94], depression [95], panic [96], aggressiveness [97], and obsessive-compulsive disorders [98].

Anthropomorphism may also lead to the owner’s misunderstanding of the dog’s feelings during supposedly positive interactions. For instance, many owners hug their dogs during affiliative interactions. However, hugging is a human expression of affection that may not be well tolerated by some dogs [99]. While some companion dogs may adapt to their owner’s manifestations of affection, others may still perceive hugging as a very invasive behavior that limits their ability to control the environment. Furthermore, hugging is often associated with the act of bending over the dog or with face-to-face proximity or contact, which may be interpreted as threatening behaviors by the animal. Therefore, it is not surprising that most dog bites in the facial region are preceded by this type of human affiliative interaction [100]. Even in the absence of an aggressive response, the dog may still display stress signals (e.g., head-turning, lip-licking, yawning, etc.) that testify to its discomfort in being forced into such interactions [101], which often occur on a daily basis. Bite prevention programs that highlight the differences between dog and human perception of affective displays and interactions may be extremely useful not just to reduce the risk of injuries [102,103,104], but also to avoid behaviors that may negatively affect dogs’ psychological well-being [101].

**Figure 6:**
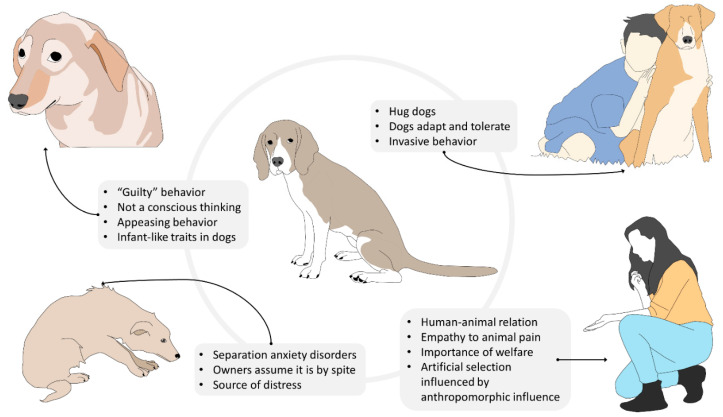
Influence of anthropomorphism on dog emotion, coping, and human–animal relations. The way humans interact and perceive animal emotions and behaviors can lead to misinterpretations of the motivation and intention of the animal, creating social consequences for them; however, when humans and their companion animal share a close bond, this can encourage empathy and the interest in their welfare.

The impact of anthropomorphism on dogs’ psychological welfare is not limited to the emotional consequences of daily inappropriate interactions with humans. In fact, for many dog breeds, the whole artificial selection process has been strongly influenced by anthropomorphic tendencies [105]. Too often, selecting dogs for physical and behavioral characteristics facilitates assigning human mental states to them that may seriously compromise dog welfare. The most infamous and evident case is that of brachycephalic breeds. In these dogs, artificial selection has focused on emphasizing human infant-like traits, such as flat faces, round cheeks, large eyes, short extremities, and even clumsiness in movements [106]. The consequence of these paedomorphic features is the emergence of the brachycephalic obstructive airways syndrome (BOAS) that compromises the efficacy of numerous vital functions, such as breathing, tissue oxygenation, thermoregulation and digestion [106,107,108], and ultimately reduces these individuals’ quality of life. Furthermore, the abnormal physical features of brachycephalic dogs may negatively affect their mimic skills and consequently compromise their ability to communicate with conspecifics [109]. Indeed, this may lead to serious intraspecific conflicts that not only jeopardize these dogs’ physical integrity but also decrease their possibilities to experience a normal and fulfilling intraspecific social life [109] (Figure 6). 

In general, dog breeds that have been selected for infant-like traits and infant-like sizes are more likely to prompt their owners to exhibit protective behaviors, exactly as a child would do with a parent. Unfortunately, some of these behaviors, such as impeding interactions with other dogs and carrying dogs around in bags or in the owner’s arms can hinder the dog’s cognitive and emotional development. These practices limit the amount of experiences dogs can make in daily life and consequently limit their ability to find strategies to cope with environmental and social stimuli. In other words, these dogs are prevented from adequately developing their skills to adapt to external changes. As a consequence, even the slightest stressor may be perceived as an insurmountable challenge. 

Furthermore, by limiting the dog’s movements, these practices affect the dog’s self-perception of control over the environment. Actual or perceived lack of control over external events has long been identified as one of the major triggers for the development of panic and anxiety disorders in both humans and companion animals [110]. 

While on the one hand anthropomorphism may foster human empathy towards non-human animals [111,112] and consequently promote a positive attitude towards animal welfare, on the other hand, it may have deleterious effects on companion animals’ emotional well-being. However, anthropomorphism is a natural tendency of humans that obligatorily shape their perception of other animal species and that cannot be completely avoided. Hence, in order to protect companion animals from extreme or deleterious anthropomorphic tendencies, education programs aimed to increase dog owners’ scientific knowledge of some easily misinterpretable dog behaviors should be more frequently implemented [113]. 

## 8. Anthropomorphism and Effects on Public Health

It is fundamental to understand the reasons why some pet owners adopt anthropomorphic practices with their pets, considering the impact these practices may have on their lives. One of the main reasons that lead people to have companion animals is the close emotional bond they develop with their pets [114]. Currently, pet animals are fundamental in many families, and various articles refer to this role as extremely positive for human psychological well-being [115]. Many different species may be defined as pets, including insects, fish, reptiles, birds, and various mammals. However, dogs and cats remain the species with which people share their lives more frequently due to their closeness with people and the empathy that humans develop for both [116]. According to Overgaauw et al. [51], around 95 and 93% of dog and cat owners, respectively, report that living with animals makes them happy, while 44% cited this as the reason for obtaining a pet. Moreover, 63% of interviewees mentioned improvements in their physical health, especially those with pet dogs because they perform more physical activity. Indeed, a whole series of physical, emotional, and health-related benefits have been attributed to pet ownership, as Table 1 shows.

Kanat-Maymon et al. [122] demonstrated that the support provided by pets represents an additional step towards greater welfare for people, though with no relation to psychological disorders; and that the human–pet interaction can be a potential origin of satisfaction of the needs for support in addition to those that come from human sources.

### Anthropomorphism and Zoonosis

Since keeping dogs and cats in people’s homes has become a routinary practice, the bonding with them has intensified over the past 50 years [123]. While the coexistence of humans and pets has grand benefits for people’s physical and emotional health [115], it is necessary to emphasize that inadequate interaction can compromise human health. In addition, dogs, in particular, play important roles in work and as companions in many cultures of developing countries and, in many nations, share the human environment [123]. Indeed, certain properly human sites and habits have been ceded to these animals, such as sharing one’s bed with a pet (usually a dog or cat), an act that 14–62% of owners allow; this means some 78 million dog owners and 95 million cat owners, according to a study carried out in the United States, the United Kingdom, France, and Holland [73]. Recently, it has become common for people to allow their pets, mainly dogs, to lick their face as a practice that demonstrates “affect”. Customs of this kind, however, have been identified as principal causes of disease transmission. In fact, reports indicate that approximately 60% of infectious diseases in humans caused by fungi, virus, and bacteria are transmitted through animals [124]. 

This panorama becomes more complicated when we consider that in certain sectors of the population, veterinary medical attention for pets is deficient or non-existent, or that owners simply do not believe their pets are sick or can carry infectious agents. This increases the risk of transmission through zoonosis [51,123], a term that refers to infectious diseases that are naturally transmissible between vertebrate animals and humans [125]. When pet owners do not understand the normal behavior of their animals, they run a greater risk of contracting a disease. For example, pets lick the anus as part of their normal biological behavior, so gastrointestinal bacterial pathogens such as *Salmonella*, *E. coli*, *Clostridium*, and *Campylobacter* can be present in their oral cavity. Allowing pets to lick the face and lips of humans is considered a potential route for transmitting these agents.

Zoonotic diseases can be of distinct origin: parasitic bacterial, and viral. To mention just two cases, bubonic plague is transmitted by the bites of fleas from infested cats that share their owner’s bed, the same that could happen with dogs. Unlike cats, dogs rarely show clinical signs of infection that could serve as alarm signals [73]. Table 2 shows the main zoonoses transmitted by pets and the anthropomorphic practices that facilitate contagion.

The permissive anthropomorphic practices that can lead to animals taking possession of their owners’ beds do not only increase the risk of contracting infectious diseases but can also create other significant risks for the physical health and integrity of, especially, people who keep dominant, possessive dogs in a bedroom where small babies sleep, for this can result in scratches, other mild lesions, and bites that, in some cases, can have fatal consequences [73], not to mention the post-traumatic stress that infants experience after being bitten [132].

A particularly irresponsible practice performed by humans that negatively impacts their own health is abandoning pets. Multitudes of stray dogs and cats walk around freely or are considered “community” animals. These animals never receive veterinary attention so they form a potential, uncontrolled reservoir of existing and new zoonotic diseases. In Nepal, for example, 95% of cases of human rabies result from contact with infected dogs, most of them strays that serve as vectors for this disease. Another factor to consider is the proximity of companion animals to wildlife. This is a cause for concern due to the risk of the direct or indirect transference of pathogens (for example, through arthropod vectors). While this association is particularly notable in animals that roam freely in the streets in rural and urban zones where they can be in contact with raccoons, opossums, urban foxes, or wild rodents [123,133].

## 9. Conclusions

Anthropomorphism is a growing tendency among human beings towards non-human animals, but its consequences can negatively affect the welfare of pets. People must understand that although pets seem to have certain similarities to human characteristics, they are not human. They must also recognize that companion animals have distinct biological needs that must be satisfied which differ by species, breed, age, physiological condition, and zootechnical aspects. As a result, understanding and recognizing that the anatomy, histology, and physiology of pets show particularities with respect to humans will help people better comprehend the commitment that humans must assume to respect the nature of their pets. 

Any action that people contemplate performing with respect to their pets must consider not only human empathy and emotions, but also be carried out on the basis of scientific evidence to avoid acts that may harm their own interests and needs. This will encourage analyses of our own physical and mental states and lead to improvements in our understanding of how human actions directly impact pet welfare, from aspects related to their biology, behavior, and habituation, to effects on our own species.

## Figures and Tables

**Table 1:** Positive effects that humans develop through interaction with companion animals.

Positive Effects of Interaction and Connections between Humans and Animals	References
Generate feelings of friendship	[73]
Hypotension	[117]
Reduced systolic and diastolic blood pressure	[118]
Reduced stress and anxiety	[117]
Effect on neurotransmitters and increased oxytocin concentrations	[117]
Provide psychological and social support	[117,119]
Reduced signology associated with depression	[117,120]
Improved self-esteem and mood, more physical exercise, and lower frequency of feelings of negativity in the face of social rejection	[117]
Increased social interaction	[117]
Sharing spare time or hours of rest as a source of psychological consolation	[73]
Minimized feelings of loneliness in older adults	[115]
Improved mental and physical health of homeless people	[121]

**Table 2:** Principal zoonoses by pets and the associated risky anthropomorphic practices.

Disease	Etiologic Agent	Principal Practice	Animal Species Involved	Signology in Humans	Reference
Cheyletiellosis	Mites: Cheyletigella: *Cheyletiella blackei* in cats, *Cheyletiella yasguri* in dogs, *Cheyletiella parasotivorax* in rabbits	Direct contact or through fomites such as floors, towels, carpets, and beds	Dogs, cats, and rabbits	Highly contagious, non-suppurating, exfoliative dermatitis, called “walking dandruff”	[54,126,127,128]
Infection	Bacteria *Staphylococcus intermedius* (common inhabitant of saliva)	Licking of ears and face	Dogs and cats	Infection in the post-mastoidectomy mastoid cavity by medium chronic otitis with cholesteatoma; Purulent sinus infection post- endoscopic resection of pituitary adenoma	[73]
Infection	Methicillin-resistant bacteria *Staphylococcus aureus*	Licking of face and sleeping with the pet	Dogs	Recurrent nasal infection	[73]
Rabia	Virus Lyssavirus, genotype 1	Bites, scratches, or licking of mucus membranes	Dogs	Lethal encephalitis	[73,129]
Toxocariasis	Parasite *Toxocara canis* *Toxocara felis*	Direct contact with eggs on the skin or hair, or with feces (kissing, licking, sleeping with pets)	Dogs and cats	Fever, cough, abdominal pain, anorexia, hepatomegaly, vision problems, lymphadenitis	[73]
Dipilidiasis	Parasite *Dipylidium caninum*	Direct contact with eggs due to ingestion of the infested flea *Ctenocephalides felis, Ctenocephalides canis* (licking or kissing the pet)	Dogs and cats	Often asymptomatic, but may show signs such as anal itching, diarrhea, moderate abdominal pain (habitually epigastric), hyporexia, indigestion, and gastrointestinal disfunctions. Occasionally, urticaria, eosinophilia, irritability, and intestinal obstruction	[130]
Infection	Pathogenic bacteria *Capnocytophaga canimorsus* (normal inhabitant of saliva)	Bites by the pet	Dogs and cats	Emergent sepsis infection, meningitis, and post-splenectomy infection after dog bites	[131]

## Data Availability

Not applicable.
